# A Nuclear-Directed Ribonuclease Variant Targets Cancer Stem Cells and Inhibits Migration and Invasion of Breast Cancer Cells

**DOI:** 10.3390/cancers13174350

**Published:** 2021-08-27

**Authors:** Jessica Castro, Giusy Tornillo, Gerardo Ceada, Beatriz Ramos-Neble, Marlon Bravo, Marc Ribó, Maria Vilanova, Matthew J. Smalley, Antoni Benito

**Affiliations:** 1 Laboratori d’Enginyeria de Proteïnes, Departament de Biologia, Facultat de Ciències, Campus de Montilivi, Universitat de Girona, Maria Aurèlia Capmany 40, 17003 Girona, Spain; gceada@ibecbarcelona.eu (G.C.); bmlopezrn@cnic.es (B.R.-N.); marlon.bravo@udg.edu (M.B.); marc.ribo@udg.edu (M.R.); maria.vilanova@udg.edu (M.V.); 2 Institut d’Investigació Biomèdica de Girona Josep Trueta (IdIBGi), 17003 Girona, Spain; 3 European Cancer Stem Cell Research Institute, School of Biosciences, Hadyn Ellis Building, Cardiff University, Cardiff CF24 4HQ, UK; tornilloG@cardiff.ac.uk (G.T.); smalleyMJ@cardiff.ac.uk (M.J.S.)

**Keywords:** anticancer drug, cancer stem cells, drug selectivity, invasion, ribonuclease

## Abstract

**Simple Summary:**

During the past decades the achievements made in treating cancers have significantly improved the survival of patients. However, cancer is still one of the leading causes of mortality. It is suggested that treatment failure is mediated by a subpopulation of tumor cells named cancer stem cells that can survive after treatment and promote cancer relapse. Targeting these cells is important to improve cancer therapy. The aim of our study is to determine the effect of a human ribonuclease variant on breast cancer cells grown in 3D and on cancer stem cells. Moreover, we study its effect on the ability of breast cancer cells to migrate and produce metastasis, responsible for about 90% of cancer deaths. We show that this ribonuclease arrests tumor cells grown in 3D without affecting normal breast cells, and this significantly inhibits cancer stem cell development. Additionally, it reduces the migratory and invasive capacities of tumor cells.

**Abstract:**

Despite the significant advances in cancer research made in recent years, this disease remains one of the leading causes of death worldwide. In part, this is due to the fact that after therapy, a subpopulation of self-renewing tumor cells can survive and promote cancer relapse, resistance to therapies and metastasis. Targeting these cancer stem cells (CSCs) is therefore essential to improve the clinical outcome of cancer patients. In this sense, multi-targeted drugs may be promising agents targeting CSC-associated multifocal effects. We have previously constructed different human pancreatic ribonuclease (RNase) variants that are cytotoxic for tumor cells due to a non-classical nuclear localization signal introduced in their sequence. These cytotoxic RNases affect the expression of multiple genes involved in deregulated metabolic and signaling pathways in cancer cells and are highly cytotoxic for multidrug-resistant tumor cell lines. Here, we show that these cytotoxic nuclear-directed RNases are highly selective for tumor cell lines grown in 3D, inhibit CSCs’ development and diminish the self-renewal capacity of the CSCs population. Moreover, these human RNase variants reduce the migration and invasiveness of highly invasive breast cancer cells and downregulate N-cadherin expression.

## 1. Introduction

Breast cancer (BC) is the second leading cause of cancer-related deaths among women worldwide [1]. The therapeutic options of the affected women are decided by evaluating a number of biomarkers: the presence of estrogen and progesterone receptors (ER and PR), an excess level of human epidermal growth factor receptor 2 (HER2) protein, and/or extra copies of the HER2 gene [2]. Using these biomarkers, BC is classified into three major molecular subtypes: hormone sensitive (ER+/PR+ or ER+/PR−, HER2−); HER2 amplified (ER−/PR− or ER+/PR−, HER2+); and triple negative (TNBC) (ER−/PR−/HER2−) [3]. TNBC is the most aggressive subtype showing an enhanced capacity of metastasis, the main cause of cancer-related deaths, and frequently leading to the appearance of multidrug resistance. It has been shown that TNBC is particularly enriched in cancer stem cells (CSCs) [4], which possess unlimited capacity for self-renewing and inherent resistance to chemotherapy. These capacities allow the post-treatment remnant cells to give rise to a differentiated cancer cell progeny, produce tumor recurrence and metastases [5,6]. Unfortunately, TNBC lacks effective therapies because conventional chemotherapy would spare the CSC population. This problem is not restricted to BC however; until now, there is no evidence of clinical success of specific CSC therapies [7]. Of note, most of the tested drugs against CSCs have defined targets affecting precise pathways but the anti-cancer activity of such “single-target” agents is most often limited against the multifactorial complexity of the malignant phenotype. Furthermore, although first-generation CSC drugs were directed against this specific sub-population, there is now an increasing consensus that an effective treatment must eradicate not only CSCs but all the transformed cells [7]. Unlike specific-targeted drugs, pleiotropic (acting on multiple targets) drugs arise as an attractive alternative since they may cope with the multifactorial complexity of stemness phenotype. In this sense, the success of combination chemotherapy over monotherapy supports this idea.

Different members of the pancreatic RNase family are selectively cytotoxic for tumor cells and have been investigated as anticancer drugs (for a review see [8]). These non-genotoxic drugs act on different RNA molecules affecting multiple pathways inside the cell and producing pleiotropic effects. An example is onconase (ONC), which reached phase II/III for the treatment of different types of cancers [9,10] but failed due to nephrotoxicity. Human pancreatic ribonuclease (HP-RNase) is considered an interesting alternative to design new anticancer RNase-based drugs because it does not show renal accumulation in vivo [11], it is not immunogenic [12] and presents higher ribonucleolytic activity [13]. We have previously developed new HP-RNase variants with antitumor activity that are routed to the nucleus [14,15,16,17] where their activity cannot be inhibited [18] and induce apoptosis [19]. These nuclear-directed RNases (ND-RNases) contain a non-contiguous extended bipartite nuclear localization signal (NLS) distributed along the sequence [20] that drives them to the nucleus, where they cleave nuclear RNA. ND-RNases are highly cytotoxic against multidrug resistant cell lines, showing synergy with doxorubicin and promoting a decrease in the expression of P-gp [21]. The characterization of the gene expression profile of ND-RNase-treated versus untreated cancer cells has shown that they affect most of the cellular pathways associated with tumorigenesis, changing the expression of genes involved in cell cycle control, apoptosis, cell signaling, tumor cell invasion and metastasis. Moreover, a reversion of the metabolic deregulated pathways in cancer cells is observed [22], confirming their pleiotropic behavior. 

Here, we have investigated the effect of one of the most active ND-RNases, NLSPE5 [15]. We show that this ND-RNase is highly selective for tumor cells grown in 3D and affects the viability of CSCs. Moreover, it inhibits migration and invasion abilities of breast cancer cells and downregulates N-cadherin expression.

## 2. Materials and Methods

### 2.1. NLSPE5 Expression and Purification

Construction of NLSPE5 has been previously described [15]. It corresponds to an HP-RNase variant carrying two nuclear localization signals (NLSs) [15]: the NLS of SV40 large T-antigen (PKKKRKVE) at the N-terminus and a conformational NLS distributed all along the sequence of the enzyme [20]. NLSPE5 was produced and purified from Escherichia coli BL21 (DE3) transformed cells essentially as described previously [23]. The molecular mass of NLSPE5 was confirmed by Matrix-assisted laser desorption/ionization time-of-flight (MALDI-TOF) mass spectrometry. NLSPE5 concentration was determined by ultraviolet spectroscopy using an extinction coefficient at 280 nm of 7950 M^−1^ cm^−1^, calculated as reported previously [24].

### 2.2. Cell Lines and Culture Conditions

Human mammary gland adenocarcinoma cell lines MCF7 and MDA-MB-231 and human breast epithelial cell line MCF10A were acquired from the American Type Culture Collection (ATCC, Rockville, MD, USA). BT474 cells (human mammary gland carcinoma cell line) were a kind gift from Dr. Anna Massaguer (University of Girona, Spain). HMEC cells (Human mammary epithelial cells) were obtained from Lonza (Basel, Switzerland).

Cells were routinely grown at 37 °C in a humidified atmosphere of 5% CO_2_. MCF7 and MDA-MB-231 cells were cultured in RPMI 1640 media (Gibco, Carlsbad, CA, USA) supplemented with 10% fetal bovine serum (FBS) (Gibco, USA), 50 U/mL penicillin and 50 μg/mL streptomycin (Gibco, USA). BT474 cell line was grown in DMEM media (Gibco, USA) supplemented with 10% FBS, 50 U/mL penicillin and 50 μg/mL streptomycin. MCF10A cells were grown in DMEM/F-12 (Gibco, USA) supplemented with 5% horse serum (Gibco, USA), 100 ng/mL cholera toxin (Sigma Aldrich, St. Louis, MO, USA), 20 ng/mL human epidermal growth factor (EGF) (Gibco, USA), 10 μg/mL insulin (Sigma Aldrich, USA), 0.5 μg/mL hydrocortisone (Sigma Aldrich, USA), 50 U/mL penicillin and 50 μg/mL streptomycin (Gibco, USA). For 3D culture experiments, cells were cultured in assay medium containing 2% horse serum (Gibco, USA), 100 ng/mL cholera toxin (Sigma Aldrich, USA), 5 ng/mL EGF (Gibco, USA), 10 μg/mL insulin (Sigma Aldrich, USA), 0.5 μg/mL hydrocortisone (Sigma Aldrich, USA), 50 U/mL penicillin and 50 μg/mL streptomycin (Gibco, USA) in DMEM/F-12 (Gibco, USA).

### 2.3. Cell Proliferation Assays in 2D

Cells were seeded into 96-well plates at the appropriate density, i.e., 4000, 2500, 20,000, 2000 and 1500 cells/well for MCF7, MDA-MB-231, BT474, MCF10A and HMEC, respectively. After 24 h of incubation, cells were treated with various concentrations of NLSPE5 for 72 h. Cell viability was determined by the CellTiter-Blue method, essentially following the manufacturer’s instructions (Promega, Madison, WI, USA). All data are described as the mean ± standard error (SE) of at least three independent experiments with three replicas in each. The IC_50_ value represents the concentration of NLSPE5 required to inhibit cell proliferation by 50%, respective to non-treated cells, and was calculated by interpolation from the obtained curves.

### 2.4. Cell Proliferation Assays in 3D

Single cells resuspended in their corresponding media containing 2.5% of Growth Factor Reduced (GFR) Matrigel™ (Corning Inc, Corning, NY, USA) were seeded into 96-well plates coated with a thin solidified layer of GFR Matrigel™. MCF7, MDA-MB-231, BT474, and HMEC, were seeded at a density of 2000, 1000, 7000 and 1000 cells/well, respectively. MCF10A cells were seeded at a density of 2000 cells/well for the short-term culture and at 650 cells/well for the long-term culture (see below). After appropriate incubation for growing on GFR Matrigel™ (i.e., 5, 2, 5 and 12 days for MCF7, MDA-MB-231, BT474 and HMEC, respectively) cells were treated with different concentrations of NLSPE5 in media containing 2.5% GFR Matrigel™ for 72 h. During the first days of growth, MCF10A single cells seeded on a basement membrane gel start to proliferate and form round spherical structures. By day 6–8, cells differentiated into a surrounding polarized layer of cells and an inner mass of less polarized cells. By day 10–15 of culture, the inner nonpolarized cells began to die by apoptosis forming a hollow lumen. These growth-arrested acini-like spheroids recapitulate many aspects of mammary architecture in vivo [25]. Taking into account that these biological events happened during MCF10A acinar morphogenesis, cells were treated with NLSPE5 for 72 h at day 5 after seeding (short-term culture) and at day 12 after seeding (long-term culture). Cell viability was determined by the CellTiter-Glo method, essentially following the manufacturer’s instructions (Promega, USA). All data are described as the mean ± SE of at least three independent experiments.

### 2.5. Mammosphere Formation Assay

Mammosphere assays were carried out in non-adherent conditions in a serum-free mammary epithelial basal medium (MEBM, Lonza, Switzerland) supplemented with 20 ng/mL EGF (Sigma Aldrich, USA), 1 μg/mL hydrocortisone (Sigma Aldrich, USA), 1 μg/mL gentamicin (Sigma Aldrich, USA), 5 μg/mL insulin (Sigma Aldrich, USA) and 2% B-27 (Invitrogen, Carlsbad, CA, USA). MCF7, BT474 and MDA-MB-231 cells were plated in ultralow attachment plates (Corning, USA) at a density of 5000 cells/mL. To examine the effect of NLSPE5 treatment on mammosphere formation, cells were seeded with and without added ND-RNase. After 7 days, primary mammospheres (P1) with diameters >50μm were manually counted and the diameter of a representative number of them was measured for every condition using an Olympus CKX41 inverted microscope and an Olympus LC30 camera. The mammosphere-forming unit (MFU) was calculated as the number of mammospheres/number of cells seeded. To evaluate the self-renewal ability of the CSC-enriched population, a second generation (P2) of mammospheres was performed. For this, P1 mammospheres were collected by centrifugation (1600 rpm, 10 min), dissociated into single-cell suspensions with 0.05% trypsin-EDTA solution (Lonza, Switzerland) and using a 23-gauge needle, and re-seeded at 5000 cells/mL in ultralow attachment plates, with no additional treatments. Seven days post-plating the MFU and the diameter of the P2 mammospheres were assessed as in passage 1. Treatment effects were described as the mean ± SE of at least three independent experiments performed in sextuplicates.

In addition, a mammosphere assay with MCF7 and MDA-MB-231 cells treated previously with NLSPE5 in monolayer cultures was carried out. For that, MCF7 and MDA-MB-231 were cultured in monolayer and treated with different concentrations of NLSPE5 for 72 h. After treatment, cells were harvested with trypsin-EDTA solution and centrifuged. Then, cells were resuspended in mammosphere medium and counted using a hemocytometer (Hausser Scientific, Horsham, PA, USA). Finally, they were seeded at 5000 cells/mL in ultralow attachment plates and at 7 days post-plating mammospheres were manually counted and their diameter was measured. The MFU was calculated as described above. All data are described as the mean ± SE of at least three independent experiments in sextuplicates.

### 2.6. Wound Closure Cell Migration Assay

MDA-MB-231 cells (6.0 × 10^5^, 2.5 mL) suspended in culture media were seeded into a 6-well plate and allowed to grow to 80% confluence. Then, a straight scratch was made using a 200 μL pipette tip, and cells were washed followed by treatment with different concentrations of NLSPE5 in media with 0.5% FBS. Images were captured at 0 h, 16 h, 24 h, 40 h and 48 h after wounding using an Olympus CKX41 inverted microscope. The wound area was quantified using the ImageJ software (National Institutes of Health, (NIH), Bethesda, MD, USA), with a wound healing tool macro (Montpellier RIO Imaging, Montpellier, France). Treatment effects were described as the mean ± SE of three independent experiments. 

### 2.7. Transwell Migration and Invasion Assays

For migration assays MDA-MB-231 cells (2.0 × 10^5^) were seeded into 6-well plates. After 24 h of incubation, cells were pretreated with various concentrations of NLSPE5 (0.05, 0.1 and 0.2 µM) for 24 h. Then, cells were trypsinized and 12,500 viable cells were suspended in 0.4 mL RPMI 1640 medium with 0.5% FBS with new treatment of NLSPE5 and seeded into the upper chamber of a 24-well transwell insert (8 µm pore size; Sarstedt, Nümbrecht, Germany). The lower chamber contained 0.5 mL RPMI 1640 medium with 10% FBS as a chemoattractant. After 48 h incubation at 37 °C, nonmigrated cells were wiped from the upper surface of the membrane with a cotton swab, and migrated cells remaining on the bottom surface were fixed with 4% paraformaldehyde for 30 min. Cells were stained with 0.2% crystal violet solution for 20 min at room temperature and ten randomly selected fields at 200× magnification were counted using an Olympus CKX41 inverted microscope. 

The invasion assays were performed by the same procedure as in the migration assay except that the number of cells seeded into the upper chamber of the transwell was 25,000 and the chamber filter was coated with GFR Matrigel™.

All data are described as the mean ± standard error SE of at least three independent experiments.

### 2.8. Tumor Spheroid-Based Migration Assays

MDA-MB-231 cells (3.5 × 10^3^, 0.2 mL) suspended in culture media with 1% GFR Matrigel™ were seeded in 96-well round bottom ultra-low attachment plates and allowed to form spheroids in suspension. After 24 h of incubation, cells were pretreated with different concentrations of NLSPE5 and further incubated for 24 h. Then, the spheroids were transferred to 96-well poly-HEMA-coated plates containing medium with 2% FBS and new treatment of NLSPE5. After 48 h of incubation, spheroids were fixed with 4% paraformaldehyde and stained with 1% crystal violet solution at room temperature. Images of the spheroids were captured using an Olympus CKx41 inverted microscope and the area covered by the migrated cells was determined using the ImageJ software (NIH, USA). Treatment effects were described as the mean ± SE of three independent experiments in triplicates.

### 2.9. Protein Sample Preparation for Western Blots

MDA-MB-231 single cells resuspended in RPMI 1640 media supplemented with 10% FBS, 50 U/mL penicillin and 50 μg/mL streptomycin and containing 2.5% of GFR Matrigel™ were seeded at a density of 15,000 cells/well into 48-well plates coated with a thin solidified layer of GFR Matrigel™. After 3 days of incubation, cells were treated with 0.5 μM NLSPE5 (a concentration equivalent to the IC30 in 3D cultures) in media containing 2.5% GFR Matrigel™ for 72 h. After treatment, wells were rinsed thoroughly with PBS complemented with a protease inhibitor cocktail (Abcam, Cambridge, UK) to ensure the removal of medium components. Then, GFR Matrigel™ was liquefied on ice by incubating for 1 h with Cell Recovery Solution (Corning, USA). Cell spheroids were collected by centrifugation and lysed using a lysis buffer (Cell Signaling Technology, Danvers, MA, USA). Cell lysates were stored at −80 °C. Protein concentrations were quantified with a Pierce BCA protein assay kit (Thermo Fisher Scientific, Waltham, MA, USA).

### 2.10. Western Blot Analysis

A total of 50 μg of protein extracts was resolved by SDS/PAGE and transferred to PVDF membranes. Membranes were probed overnight with primary antibodies (Santa Cruz Biotechnology, Dallas, TX, USA) directed against the following proteins: N-cadherin (sc-393933), E-cadherin (sc-8426), MMP-2 (sc-13595), MMP-9 (sc-21733) and actin (sc-8432). The secondary antibody (mouse anti-rabbit peroxidase conjugate (sc-2357, Santa Cruz Biotechnology) was incubated for 1 h at room temperature. Blots were developed with immobilon chemiluminescent HRP substrate (Millipore, Bedford, MA, USA) and images were captured by a FluorChem SP system (Alpha Innotech, San Leandro, CA, USA). Quantity analysis is based on the intensity of the band using the ImageJ software (NIH, USA).

### 2.11. Statistical Analysis

Statistical analysis was carried out using IBM SPSS 24 software for Windows (Armonk, NY, USA). T-tests and one-way ANOVA followed by a Dunnett post hoc test were performed, taking a significance level of 0.05.

## 3. Results

### 3.1. NLSPE5 Is More Cytotoxic for Cancer Cells Than Normal Cells in 3D Culture

Firstly, we investigated the effect of NLSPE5 on a panel of three BC cell lines, representative of different subtypes of BC, and on two non-tumor breast cell lines, cultured in 2D. In these conditions, NLSPE5 is highly cytotoxic against MDA-MB-231 and MCF7 cell lines but much less cytotoxic for BT474 cell line (Table 1). Regarding the non-tumor cells, the cytotoxicity of NLSPE5 on the human mammary epithelial cells HMEC was similar to that observed for the MDA-MB-231 and MCF7 cell lines, whereas the IC_50_ of the human breast epithelial cell line MCF10A was nearly 4-fold higher (Table 1). 

**Figure 1:**
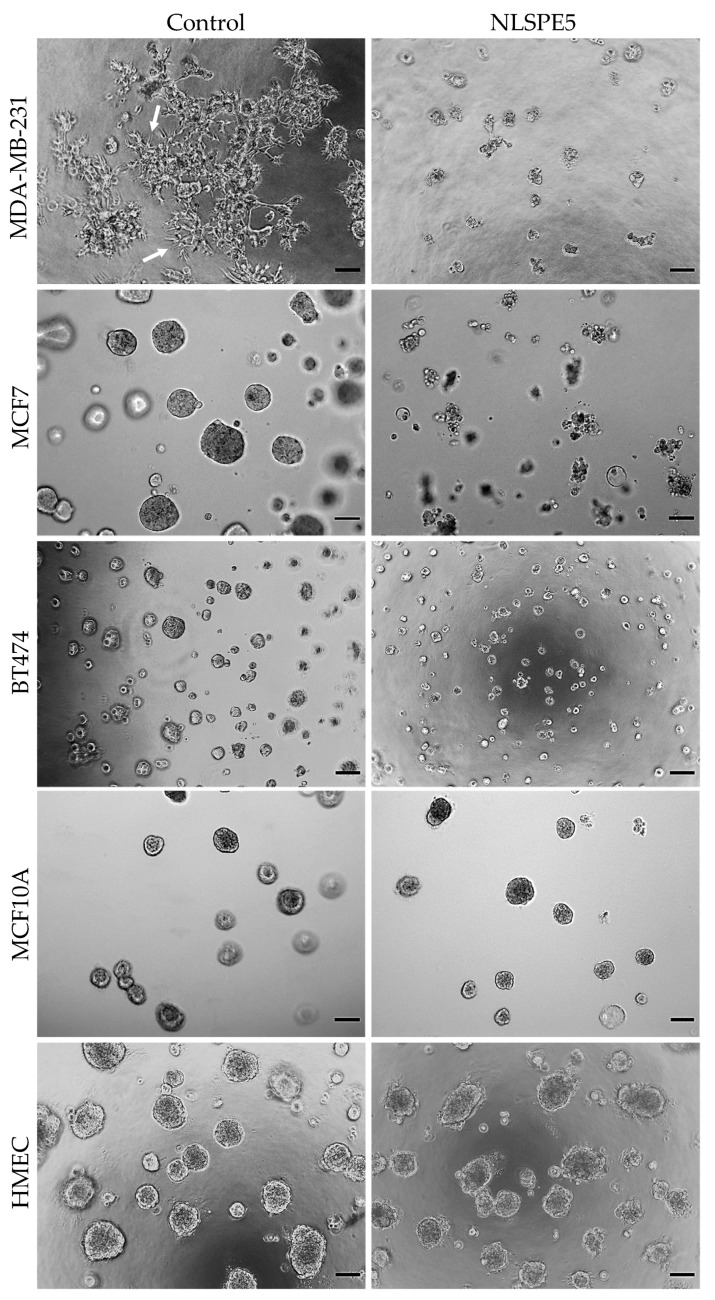
Representative images of 3D cultures treated with NLSPE5 (20 µM for tumor cell lines and 30 µM for non-tumor cells). MDA-MB-231, MCF7, BT474, MCF10A and HMEC cells seeded on GFR Matrigel™ formed spheroids. Once the spheroids formed, they were treated with NLSPE5 and incubated for 3 days before measuring the cell viability. Arrows indicate cells with a stellate form. Scale bar 100 μm.

The cytotoxicity of NLSPE5 was then determined on breast cancer and normal breast cells grown in three-dimensional (3D) laminin-rich extracellular matrix cultures in order to better recapitulate in vivo responses to NLSPE5 [26,27,28]. Important differences were observed when cells were grown in 3D: MCF7 and BT474 cancer cell lines formed round mass colonies on top of matrix while MDA-MB-231 cancer cells acquired a stellate form, growing as branching tubular structures with processes emanating from them. Conversely, MCF10A and HMEC formed acinar spheroids (Figure 1). 

NLSPE5 remained cytotoxic for MCF7 and MDA-MB-231 cells although IC_50_ values increased in 3D respect to 2D. The effect of NLSPE5 on BT474 cells was much lower, both in 2D and 3D (Table 1). Interestingly, a clear decrease in the stellate prolongations was observed in MDA-MB-231 cells upon NLSPE5 treatment in 3D (Figure 1). According to the temporal progression of the development of the acinar structures in MCF10A [25], cells were treated with NLSPE5 after 5 and 12 days of culture in 3D and a notably increase in the IC_50_ was observed in both cases with respect to the values obtained for the tumor cell lines MCF7 and MDA-MB-231 (Table 1). The same results were obtained when the primary mammary epithelial cells HMEC were treated with NLSPE5 after 12 days of culture in GFR Matrigel™ (Table 1). Therefore, while tumor cells remain rather sensitive to NLSPE5 in 3D culture conditions, normal cells become more resistant and are somehow protected in 3D. The results indicate that NLSPE5 target effectively breast cancer cells with no or minimal effects on normal cells in 3D culture conditions.

### 3.2. NLSPE5 Decreases the Capacity of Formation of Mammospheres and Their Diameter in BC Cell Lines

**Figure 2:**
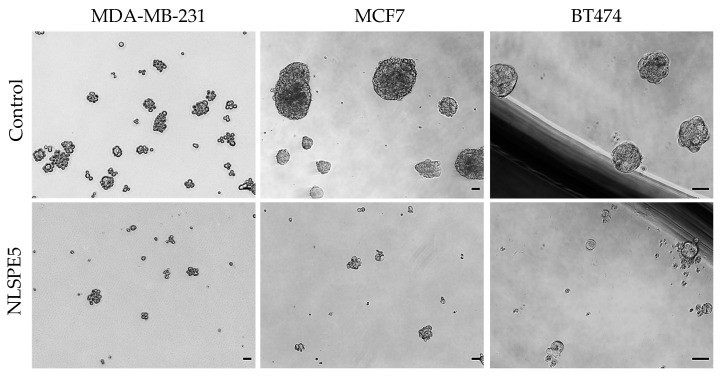
Representative images of mammospheres. MDA-MB-231, MCF7 and BT474 cell lines were treated in suspension with NLSPE5, prior to be seeded in ultralow attachment plates, and incubated for 7 days (passage 1). Scale bar 50 μm.

Spontaneous mammosphere formation in ultralow attachment plates was performed for the evaluation of NLSPE5 efficacy against CSCs. All the BC cell lines assayed (i.e., MCF7, MDA-MB-231, and BT474) were able to form mammospheres in ultralow attachment plates. MCF7 and BT474 cells formed big compact mammospheres, while MDA-MB-231 cells formed mammospheres with a smaller number of cells (Figure 2) as already reported [29]. NLSPE5 affected all the BC cell lines seeded in ultralow attachment plates and treated with the ND-RNase in suspension conditions (Figure 2). 

**Figure 3:**
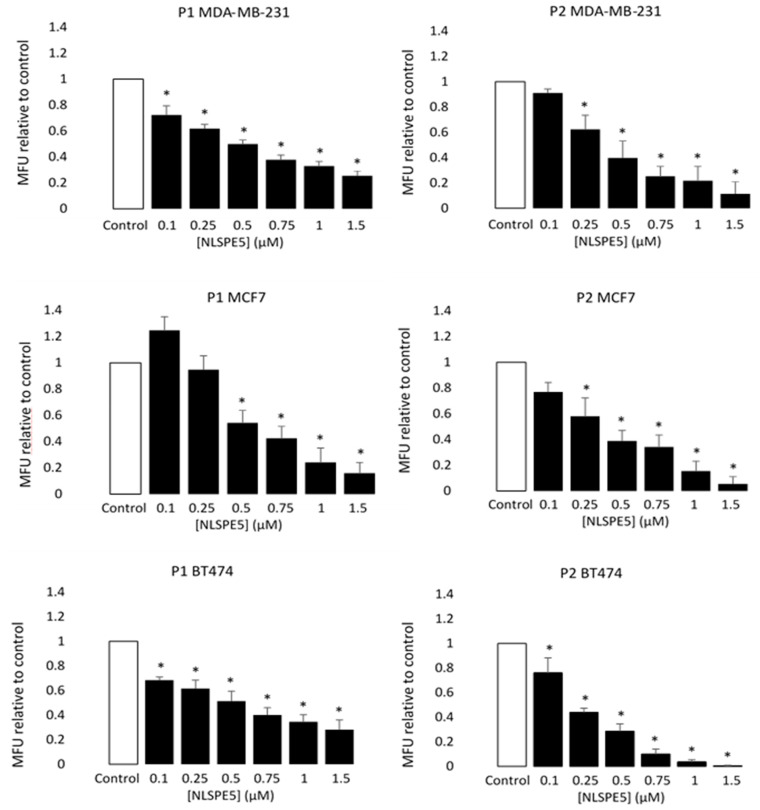
Cytotoxicity of NLSPE5 against CSCs. MDA-MB-231, MCF7 and BT474 tumor cells were seeded in ultralow attachment plates and treated with different concentrations of NLSPE5. Mammosphere number was measured at the first (P1) and second (P2) generation. MFU was calculated as detailed in the materials and methods section and the MFU of every condition was normalized to the MFU of the control. Data are presented as mean ± SE of at least three independent experiments performed in sextuplicates. Differences versus untreated control cells were considered significant at * *p* < 0.05.

When MCF7, BT474 and MDA-MB-231 cancer cells were treated with NLSPE5, the mammosphere forming unit (MFU; i.e., the number of cells seeded capable of forming mammospheres) decreased for all the tumor cell lines assayed and in both passages 1 and 2 in a dose-dependent manner (Figure 3). These data indicate that NLSPE5 affects self-renewal of CSCs. 

**Figure 4:**
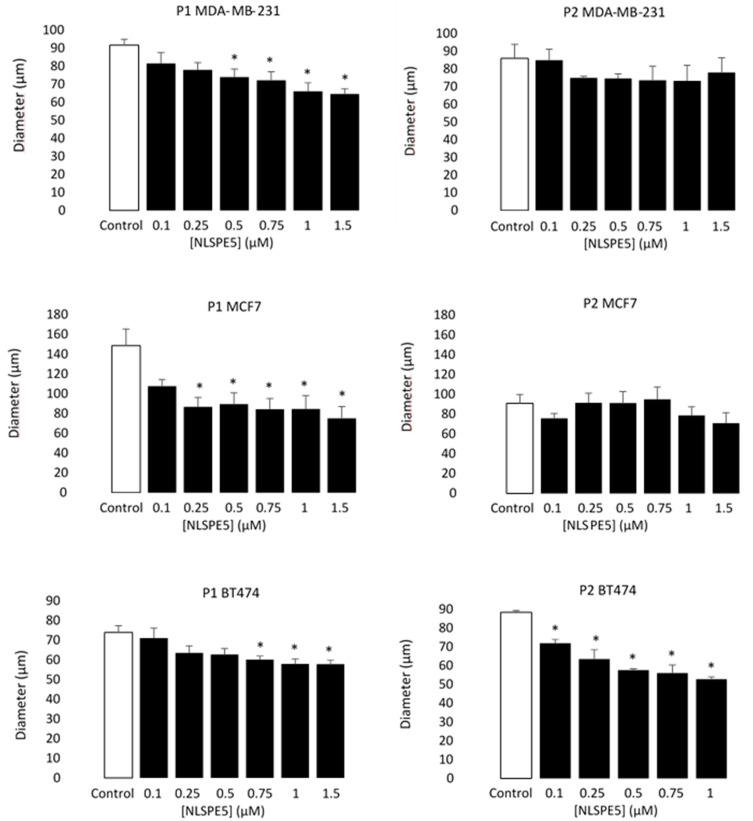
Antiproliferative effect of NLSPE5 against CSCs. Diameter of a representative sample of MDA-MD-231, MCF7 and BT474 mammospheres was measured at the first (P1) and second (P2) generation. Data are presented as mean ± SE of at least three independent experiments performed in sextuplicates. Differences versus untreated control cells were considered significant at * *p* < 0.05.

Moreover, the diameter of the mammospheres decreased in respect to the control in all cell lines at passage 1 (P1) when NLSPE5 was present in the media, while at passage 2 (P2), significant differences in diameter were observed only in BT474 cells (Figure 4).

**Figure 5:**
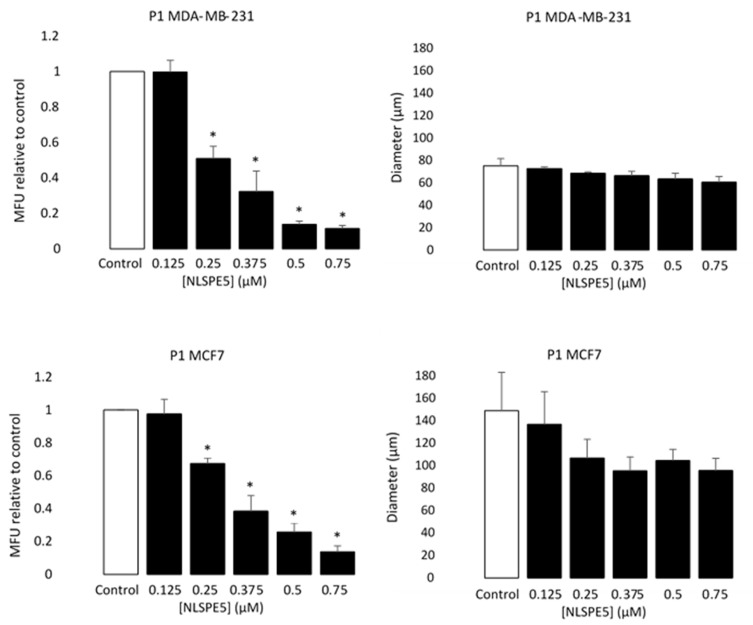
Selectivity of NLSPE5 against CSCs. MDA-MD-231 and MCF7 tumor cells were treated in monolayer cultures with different concentrations of NLSPE5 and after 3 days of incubation seeded in ultralow attachment plates. Mammospheres number and diameter of a representative sample was measured at the first (P1) generation. MFU was calculated as detailed in Materials and methods section and the MFU of every condition was normalized to the MFU of the control. Data are presented as mean ± SE of at least three independent experiments performed in sextuplicates. Differences versus untreated control cells were considered significant at * *p* < 0.05.

On the other hand, MCF7 and MDA-MB-231 cells were cultured and treated in monolayer with NLSPE5, and after 3 days of incubation were seeded in ultralow attachment plates. In this case, since the RNase was not present in the media, no significant differences in the diameter of the mammospheres were observed (Figure 5). However, the effect of NLSPE5 on the number of MFU was even higher than that observed when the cells were treated in suspension as described above (Figure 5), suggesting that NLSPE5 indeed depletes the CSCs pool in these cell lines.

### 3.3. NLSPE5 Inhibits the Migration and Invasion of the BC Cells and Downregulates N-Cadherin Expression

**Figure 6:**
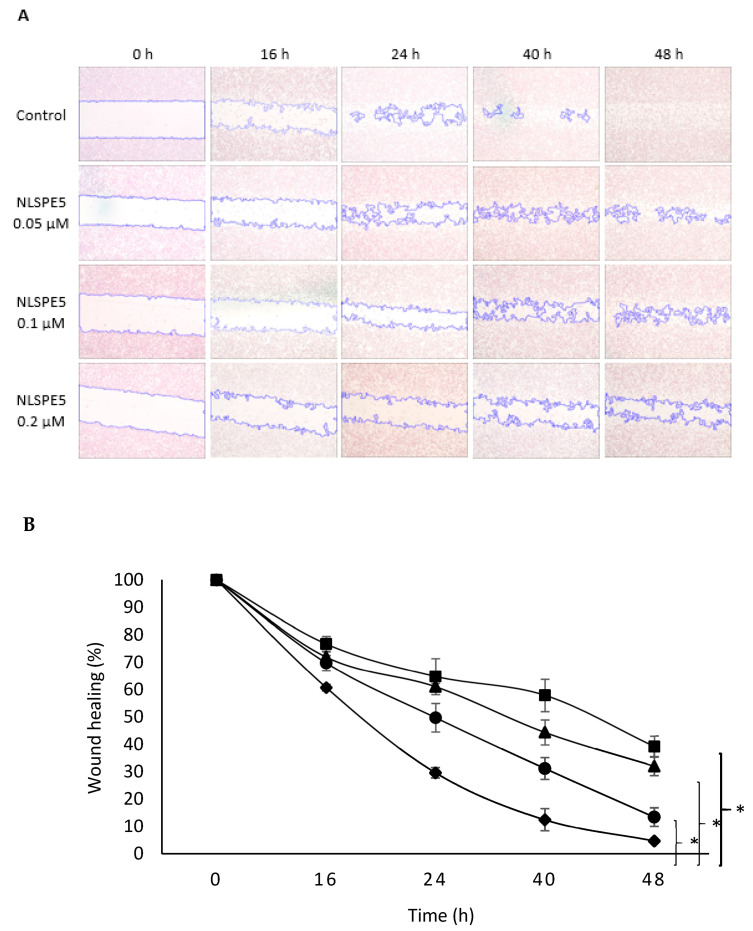
Effect of NLSPE5 on cell migration by wound closure assay. (**A**) Representative images from wound healing assay of MDA-MB-231 cell cultures untreated and treated with NLSPE5 at 0, 16, 24, 40 and 48 h. (**B**) Summary graph illustrating percentage wound healing at indicated time points of MDA-MB-231 cell cultures untreated (black diamond) and treated with NLSPE5 at 0.05 µM (black circle), 0.1 µM (black triangle) and 0.2 µM (black square). * *p* < 0.05. Data are shown as mean ± SE of three independent experiments.

CSCs are the most aggressive cell population within the tumor, and they are considered to be largely responsible for cell migration and invasion [30]. To measure the effect of NLSPE5 on cell migration, wound closure, transwell analysis and tumor spheroid-based migration assays were performed. Regarding the wound closure experiments, the untreated control cells exhibited marked cell migration in the wounded area, whereas the wounds of cells treated with NLSPE5 showed an important delayed healing at all the time points assayed (16 h, 24 h, 40 h and 48 h) in a dose-dependent manner (Figure 6). 

**Figure 7:**
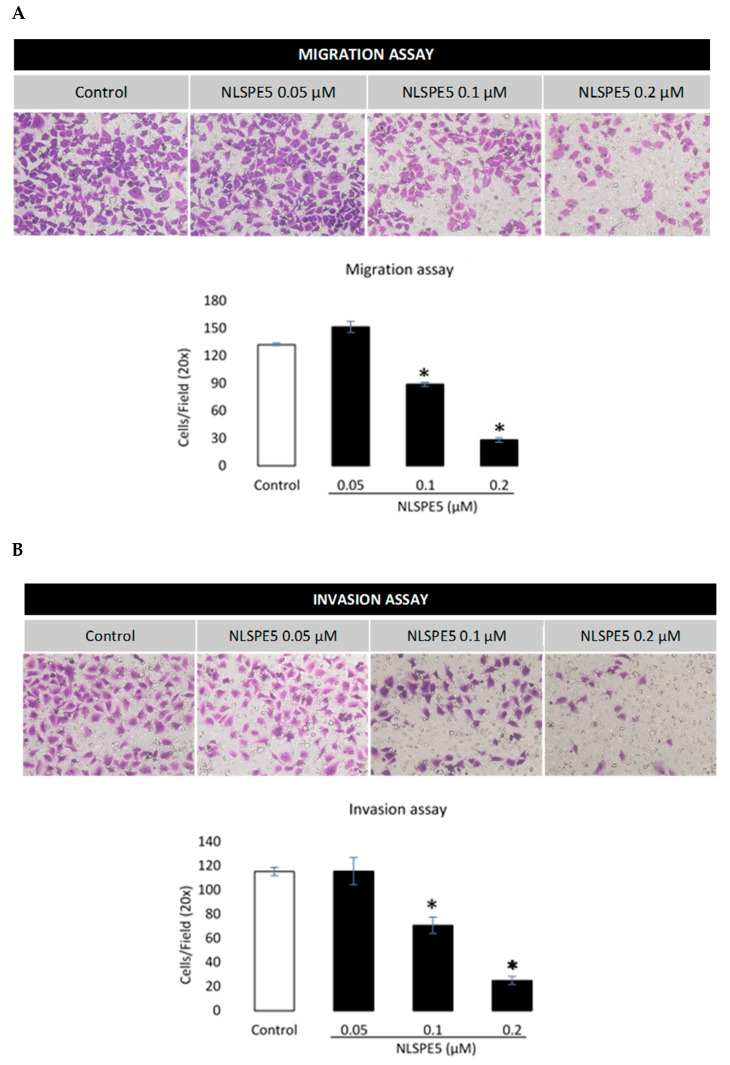
Effect of NLSPE5 on migration and invasion of MDA-MB-231 cells using a transwell system. (**A**) Representative images of migration assays in cells untreated and treated with NLSPE5 for 72 h and quantitative analysis of cell density. (**B**) Representative images of invasion assays of cells untreated and treated with NLSPE5 for 72 h and quantitative analysis of cell density. Cells were stained purple with crystal violet. * *p* < 0.05. Data are shown as mean ± SE of at least three independent experiments.

**Figure 8:**
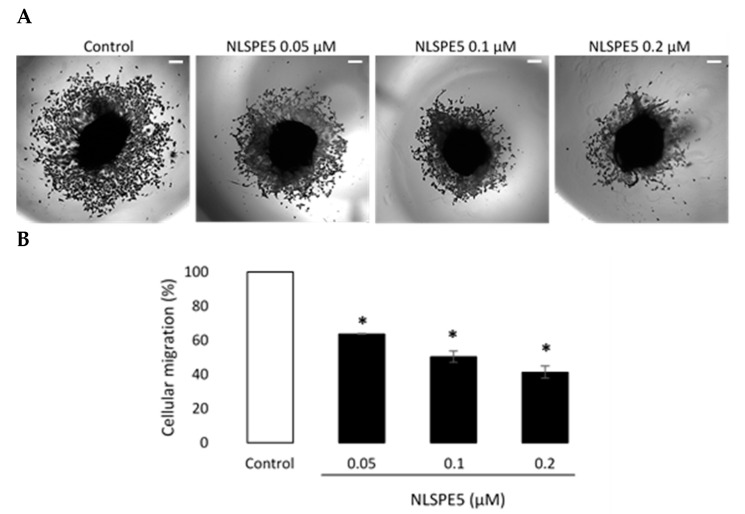
Three-dimensional tumor spheroid-based migration assay. (**A**) Representative images of migration assays in MDA-MB-231 cells untreated and treated with NLSPE5 at the indicated concentrations, obtained using a conventional inverted microscope. (**B**) Quantification of tumor cell migration. * *p* < 0.05. Data are shown as mean ± SE of three independent experiments performed in triplicates. Scale bar 200 μm.

In addition, NLSPE5 significantly reduced the migration of BC cells in transwell assays at 0.1 µM and 0.2 µM after 72 h of incubation (Figure 7A). When the effect of NLSPE5 on migration of BC cells was tested in a more realistic approach to the behavior in vivo using a 3D cancer cell spheroid migration assay, NLSPE5 significantly inhibited the migration at all the concentrations assayed. In this assay, even the lowest dose of NLSPE5 impaired the migration of BC cells (Figure 8).

The effect of NLSPE5 on invasion of BC cells was tested in a transwell assay with GFR Matrigel™. Compared with the control group, NLSPE5-treated cells showed a significantly lower capacity of invasion at 0.1 µM and 0.2 µM after 72 h of incubation (Figure 7B).

**Figure 9:**
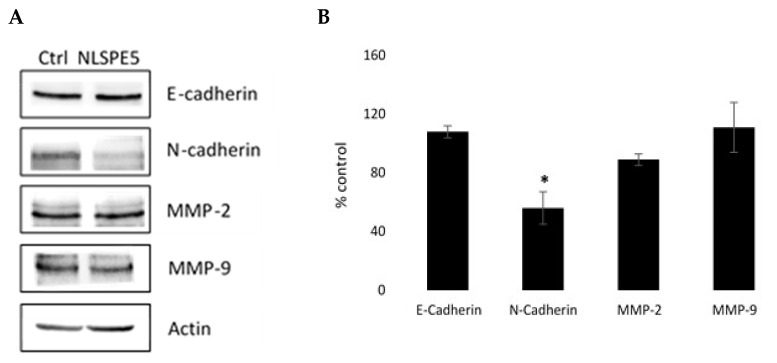
Western blot analysis of the expression of different proteins involved in the migratory and invasive capacities of tumor cells. (**A**) Representative Western blots. (**B**) Expression levels relative to that of actin. Values of untreated cells are considered as 100%. Differences versus untreated control cells were considered significant at * *p* < 0.05. Original blots can be found at Figure S1.

Additionally, through Western blotting, we investigated the effect of NLSPE5 on the expression of N- cadherin and E-cadherin and the matrix metalloproteinases MMP-2 and MMP-9 in MDA-MB-231 cells, which are involved in the migratory and invasive capacities of tumor cells. These assays showed that NLSPE5-treated cells significantly downregulated the expression of N-cadherin (Figure 9). Immunoblotting also showed that the treatment with NLSPE5 does not significantly alter the expression of E-cadherin and the metalloproteases MMP-2 and MMP-9 (Figure 9).

## 4. Discussion

We had previously shown that NLSPE5 is cytotoxic for many different tumor cell lines of different origin in two-dimensional (2D) cultures [15]. Since these cultures fail to recapitulate the physiology of a tumor tissue and the conditions that mimic the multicellular architecture and cell relationships that occur in vivo, we investigated the effect of NLSPE5 in 3D cultures, which exhibit features that are closer to the in vivo conditions [31]. In these studies, three tumor cell lines representative of different BC subtypes were chosen: MCF7 (a luminal A subtype BC cell line) [32,33,34], BT474 (a luminal B subtype BC cell line) [35] and MDA-MB-231 (a highly metastatic TNBC subtype cell line) [36,37]. Different cellular subpopulations have been shown to exist in growing cultures of MCF7, one of them corresponding to CSCs capable of regenerating the remaining subtypes [38]. The HER2-positive cell line BT474 also contain CSCs [39] and MDA-MB-231 cell line is enriched for markers associated with the epithelial-mesenchymal transition (EMT) and the expression of features associated with mammary CSCs, such as the CD44+CD24−/low phenotype [40]. 

We show that NLSPE5 is more cytotoxic for MCF7 and MDA-MB-231 cell lines in both 2D and 3D cultures. In contrast, BT474 cells are less sensitive to the effects of NLSPE5, showing cytotoxicity values about 30 times lower. 

The IC_50_ values for 3D cultures are somehow lower, but this observation has been already reported in different studies [41,42]. Sometimes, this fact has been attributed primarily to signals from dynamic cellular interactions between neighboring cells and ECM input into the cellular decision-making process [43], but could also be due to a lower drug uptake through the spheroid structure and to hypoxia, which can lead to the activation of genes involved in cell survival and drug sensitivity [44]. 

The selectivity of NLSPE5 against BC cell lines has been investigated by comparing its cytotoxicity on tumor cells with that obtained on non-tumor cells: the breast epithelial cell line MCF10A and the human mammary epithelial cells HMEC. MCF10A is a spontaneously immortalized, but non-transformed human mammary epithelial cell line derived from fibrocystic mammary tissue. These latter cells display numerous characteristics of normal breast epithelium, including lack of tumorigenicity in nude mice, dependence on growth factors and hormones for proliferation and survival and lack of anchorage-independent growth [45].

In 2D cultures, the IC_50_ values are similar to those obtained for MCF7 and MDA-MB-231 cell lines. It has been shown that cells cultured in 2D exhibit distinct differences in cell metabolism, resistance to apoptosis and responsiveness to drug treatment when comparing in vivo environment [46,47]. Various studies have shown that cells cultured in 2D are under stress and some genes and proteins expressed are altered due to their unnatural state. These gene expression alterations play a major role in drug sensitivity [27] and could explain these low IC_50_ values. Nevertheless, when MCF10A and HMEC normal cells are cultured in 3D and form acinar spheroids, an architecture similar to that observed in the mammary gland in vivo [25], the IC_50_ increases notably, indicating that NLSPE5 display a clear selective cytotoxicity for tumor cells cultured in 3D. This increase could be explained because when normal cells are cultured in 3D they recover their tissue-specific architecture as well as their mechanical and biochemical characteristics, which can lead to the normal gene expression of the genes involved in cell survival and drug sensitivity. Since in vitro 3D cell models are a good predictor of in vivo drug responses [26,27,28], these results suggest that treatment with NLSPE5 in vivo might have limited toxicity on normal tissues.

The mammosphere formation assay in ultralow attachment plates has allowed us to evaluate the cytotoxicity of NLSPE5 against CSCs. This approach is based on the observation that when a single-cell suspension is seeded in serum-free media containing growth factors and plated in ultralow attachment plates, only stem cells can form spheres [48]. When MCF7, BT474 and MDA-MB-231 cancer cells were treated with NLSPE5 in ultralow attachment plates, the MFU decreased for all the tumor cell lines assayed and in both passages 1 and 2, inhibiting CSC development and diminishing the self-renewing property of the CSC population. Therefore, even though BT474s are less sensitive to the cytotoxic effects of NLSPE5, it still inhibits their mammosphere-forming potential. Additionally, when MCF7 and MDA-MB-231 cells were treated first in monolayer with NLSPE5, the MFU decreased even more than when the cells were treated in suspension, showing an apparent selectivity for CSCs in both cell lines. Moreover, the diameter of the mammospheres, which is a measure of the proliferative potential of the cells, decreased when NLSPE5 was present in the media. Therefore, NLSPE5 is decreasing the proliferation of the cells, which is in concordance with the cytostatic effect of its parental variant PE5 [19]. Nevertheless, in the case of BT474 cells, the apparent discrepancy between the IC_50_ values obtained by CellTiter assays and the decrease in the diameter of the mammospheres treated with the ND-RNase, considering that the two methods measure proliferation, could be due to a reduction in the size of the treated cells rather than a reduction in the number of cells forming the spheres. 

Cell migration is a key hallmark of malignant cells that contributes to the progression of cancers from a primary, localized mass to an invasive and/or metastatic phenotype. Accordingly, there is currently a pressing need for novel drugs that target processes central to metastatic disease including migration and invasion of cancer cells. We demonstrate here that NLSPE5 effectively reduces the migration and invasiveness of the highly invasive BC cell line MDA-MB-231 and that it decreases the expression of N-cadherin. The 3D tumor spheroid-based migration assay, which more accurately reflects the solid tumor microenvironment and can accommodate both extracellular matrix and host cell interactions [49], corroborates this antimigration effect. N-cadherin plays a central role in tumor cell migration and invasion. Expression of N-cadherin increases tumor cell motility in many types of cancers such as breast, prostate, and gastric cancer [50]. Moreover, N-cadherin promotes tumor cell adhesion to extracellular matrix and endothelium, playing a key role in tumor metastasis [51,52]. It has also been reported that interfering with N-cadherin expression is sufficient to prolong survival in a highly metastatic pancreatic cancer model [53]. Overall, the N-cadherin downregulation observed after the treatment with NLSPE5 could explain at the molecular level, at least in part, the inhibitory effect of NLSPE5 on the migration and invasiveness of breast cancer cells.

## 5. Conclusions

Conventional anticancer therapies abolish the bulk of proliferating tumor cells, but after treatment a subset of cells can survive, promoting tumor relapse due to their ability to establish higher invasiveness and resistance to therapies. Therefore, drugs with pleiotropic effects such as RNases that target multiple forms of RNA can cope with the complex cancer physiology, and thus are interesting candidates for use in cancer therapy. In this paper, we show that the nuclear-directed ribonuclease variant NLSPE5 displays a high selectivity for tumor cells grown in 3D culture and significantly decreases the viability of CSCs. Moreover, it notably reduces the migration and invasiveness of breast cancer cells and downregulates N-cadherin expression.

## Figures and Tables

**Table 1:** IC_50_ values (µM) of NLSPE5 treatment of tumor and non-tumor cells cultured in 2D and 3D.

Variation	2D	3D	IC_50_ 3D/IC_50_ 2D *
MCF7	0.64 ± 0.08	1.09 ± 0.05	1.7
MDA-MB-231	0.39 ± 0.07	1.32 ± 0.16	3.4
BT474	17.0 ± 1.53	29.29 ± 4.17	1.7
MCF10A	1.50 ± 0.09	>30 (day 5) >30 (day 12)	>20 >20
HMEC	0.35 ± 0.10	>30 (day 12)	>85.7

* IC_50_ values correspond to the concentrations of NLSPE5 that are required to inhibit cell proliferation by 50%. Data are presented as mean ± SE of at least three independent experiments.

## Data Availability

The data presented in this study are available on request from the corresponding author.

## Supplementary Materials

The following are available online at https://www.mdpi.com/article/10.3390/cancers13174350/s1, Figure S1: original western blots.
