# Supplementary material for: A Nuclear-Directed Ribonuclease Variant Targets Cancer Stem Cells and Inhibits Migration and Invasion of Breast Cancer Cells
Source: Cancers (Basel). 2021 Aug 27;13(17):4350. doi: 10.3390/cancers13174350 (PMC8430808; doi:10.3390/cancers13174350)

Figure S1. Western blot analysis of the expression of different proteins involved in the migratory and invasive capacities of tumor cells. For each blot the used antibodies are indicated. Lane 1: control. Lane: NLSPE5 treated cells.

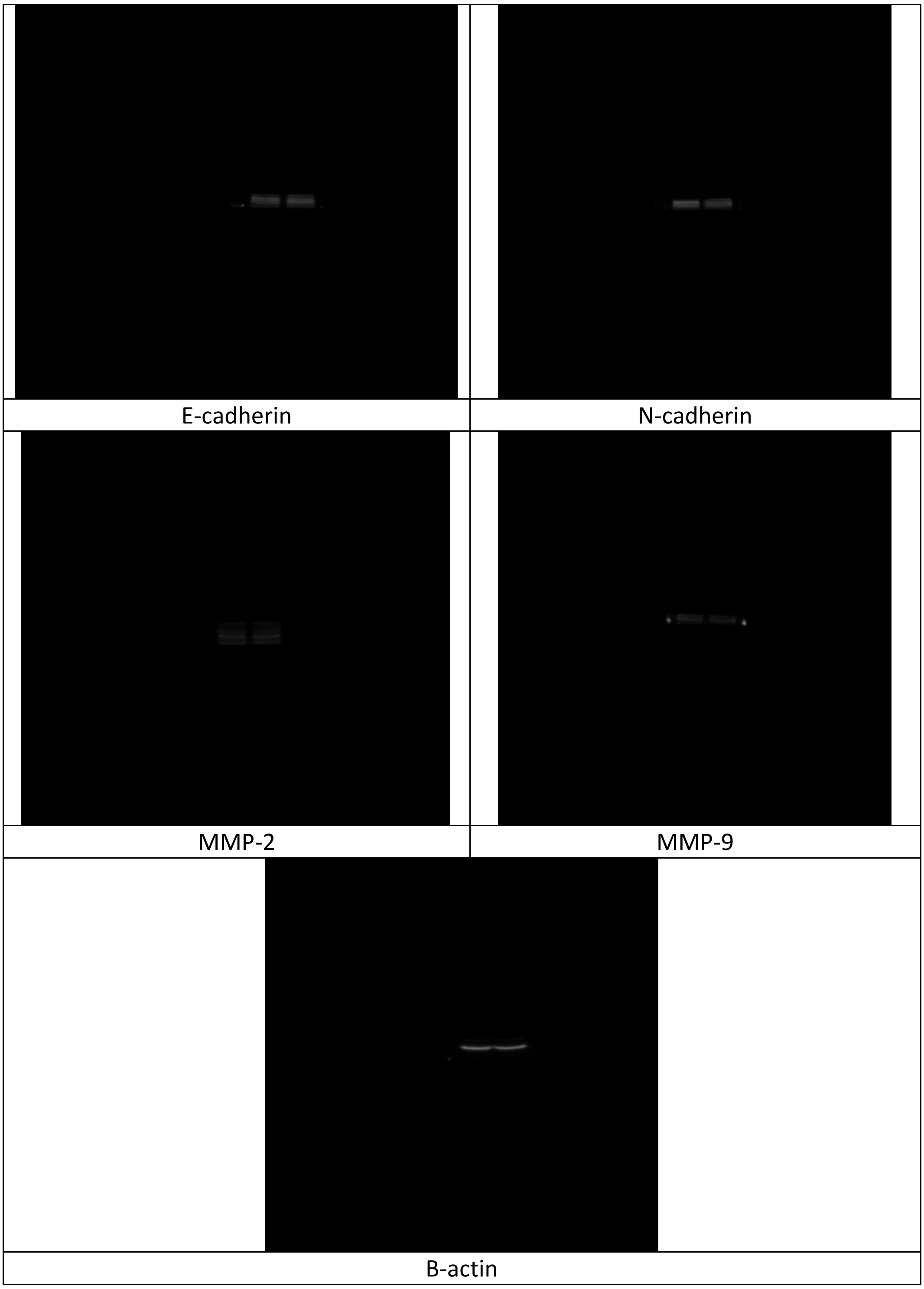

Supplement: Supplementary file 1 [file cancers-13-04350-s001.zip › cancers-1346056-supplementary.pdf]
